# Lamin B1 affects nuclear shape and integrity through chromatin stiffness and not lamin stiffness

**DOI:** 10.64898/2026.08.10.744010

**Published:** 2026-08-11

**Authors:** Andy Li, Catherine G Chu, Nick Lang, Edward J Banigan, Andrew Daniel Stephens

## Abstract

The mechanical properties of the nucleus are critical for maintaining nuclear integrity and function. We previously showed that chromatin dominates short-extension mechanics whereas lamins provide long-extension strain stiffening. To distinguish the roles of lamin isoforms, micromanipulation nucleus force measurements were performed on isolated nuclei from lamin A/C (Lmna-/-) and lamin B1 (Lmnb1-/-) knockout mouse embryonic fibroblast cells. Lamin A/C knockout does not alter short-extension nuclear stiffness but is essential for strain stiffening at longer extensions. Oppositely, lamin B1 loss reduced short-extension stiffness due to facultative heterochromatin loss while long-extension strain stiffening was slightly increased. Loss of lamin A/C and B1 resulted in similar lamin-chromatin linkers effects as LBR did not change and LAP2β decreased in both. A simulation model of a polymeric lamin shell with stiff lamin A/C and softer lamin B1 subunits can qualitatively recapitulate experimental measurements of lamin knockout cells. Lamin A/C knockout resulted in abnormal nuclear shape but not nuclear blebbing or rupture whereas lamin B1 knockout, similar to other perturbations that cause heterochromatin loss, resulted in increased nuclear blebbing and rupture. This work illuminates the distinct mechanical roles of lamin A/C and B1 in determining nuclear structure and integrity.

## Full Text Availability

The license terms selected by the author(s) for this preprint version do not permit archiving in PMC. The full text is available from the preprint server.

